# Physics-informed shape optimization using coordinate projection

**DOI:** 10.1038/s41598-024-57137-4

**Published:** 2024-03-19

**Authors:** Zhizhou Zhang, Chungwei Lin, Bingnan Wang

**Affiliations:** Mitsubishi Electric Research Laboratories, 201 Broadway, 8th Floor, Cambridge, MA 02139-1955 USA

**Keywords:** Physics-informed neural network, Shape optimization, Coordinate projection, Weak formulation, Applied physics, Theory and computation, Computational science, Applied physics, Theory and computation, Computational science

## Abstract

The rapid growth of artificial intelligence is revolutionizing classical engineering society, offering novel approaches to material and structural design and analysis. Among various scientific machine learning techniques, physics-informed neural network (PINN) has been one of the most researched subjects, for its ability to incorporate physics prior knowledge into model training. However, the intrinsic continuity requirement of PINN demands the adoption of domain decomposition when multiple materials with distinct properties exist. This greatly complicates the gradient computation of design features, restricting the application of PINN to structural shape optimization. To address this, we present a novel framework that employs neural network coordinate projection for shape optimization within PINN. This technique allows for direct mapping from a standard shape to its optimal counterpart, optimizing the design objective without the need for traditional transition functions or the definition of intermediate material properties. Our method demonstrates a high degree of adaptability, allowing the incorporation of diverse constraints and objectives directly as training penalties. The proposed approach is tested on magnetostatic problems for iron core shape optimization, a scenario typically plagued by the high permeability contrast between materials. Validation with finite-element analysis confirms the accuracy and efficiency of our approach. The results highlight the framework’s capability as a viable tool for shape optimization in complex material design tasks.

## Introduction

Recent advances in large foundation artificial intelligence (AI) models have demonstrated their potential in addressing intricate real-world challenges^1,2^, consequently drawing an increasing number of researchers to apply AI to scientific problems, such as carbon capture^3,4^, weather forecast^5,6^, material discovery^7,8^, simulation acceleration^9,10^, etc. One major characteristic that distinguishes scientific problems from other AI tasks is the existence of prior knowledge. Prior knowledge can manifest in various forms in scientific problems, and may significantly enhance the performance of ML models, particularly when ground truth data is insufficient^11,12^. A prime example is the incorporation of governing partial differential equations (PDEs) through PINN, into the training of machine learning (ML) models for predicting engineering problems^13,14^.

Originally proposed as a forward solver to PDEs, PINN has been receiving growing research attention recently, for its data-free self-supervised training process^14,15^. The major advantages of PINN over classical numerical methods include mesh-free representation, higher parameter efficiency in high dimensional systems, general and concise training formulation^13,16,17^. Researchers have implemented PINN to solve PDEs in various real-world engineering systems including solid mechanics^18^, fluid mechanics^16^, thermodynamics^19^, electromagnetism^20^, etc. On the other hand, the following disadvantages of PINN still impede its use in industry, and pose the necessity of further exploration: optimization error, intractable integral (can only be approximated)^16,21^. Most importantly, empirical studies suggest that employing PINN as PDE solvers can introduce considerable computational costs, both in terms of memory space and processing time, when compared to classical numerical methods^22^.

Despite its subtle performance as a PDE solver, physics-informed training strategy shows greater potential in design exploration tasks, as it converts an equation solving process into an optimization problem. Compared to traditional optimization algorithms, for instance adjoint method based sensitivity analysis^23^, physics-informed design optimization is significantly more general and easier for adaptation. Cutting-edge research progress in physics-informed design optimization concentrates on two main directions. The first direction is to establish a surrogate ML model that learns a response function for the parameterized shape or topology design space^24–28^. A well-trained surrogate model can typically accelerate the PDE solution process by at least $3-4$ orders of magnitude, with negligible prediction error^18,29^. Recent efforts have discovered novel neural operator architectures that allow projection among infinite-dimensional function spaces^30,31^. The input space of these architectures possesses discretization-invariance and is inherently closer to physics fields, thus achieving higher prediction accuracy when fed with sufficient data. However, surrogate-model based methods assume relatively simple geometry parameterization that is concise enough to be included as part of the model inputs. This assumption becomes invalid for general shape representations that may involve hundreds of thousands of parameters. On the other hand, the second research direction focuses on direct optimization of some parameterized property field^32,33^. The design space (property field) is typically parameterized explicitly as a density field^34–38^ or implicitly as a level-set function^39–41^. These parameterization techniques can be applied to numerous design tasks. This type of method assumes continuity and differentiability of the material density distribution over space and thus introduces transition regions across subdomain interfaces, which may generate inaccurate physics solutions when large material property gaps exist (an example shown in SI Appendix). This inaccurate approximation due to material field smoothing becomes particularly challenging when neural networks are utilized to parameterize material property fields or physics fields, as neural networks are highly smooth, and sometimes possess an infinite degree of differentiability like sinusoidal waves.

**Figure 1:**
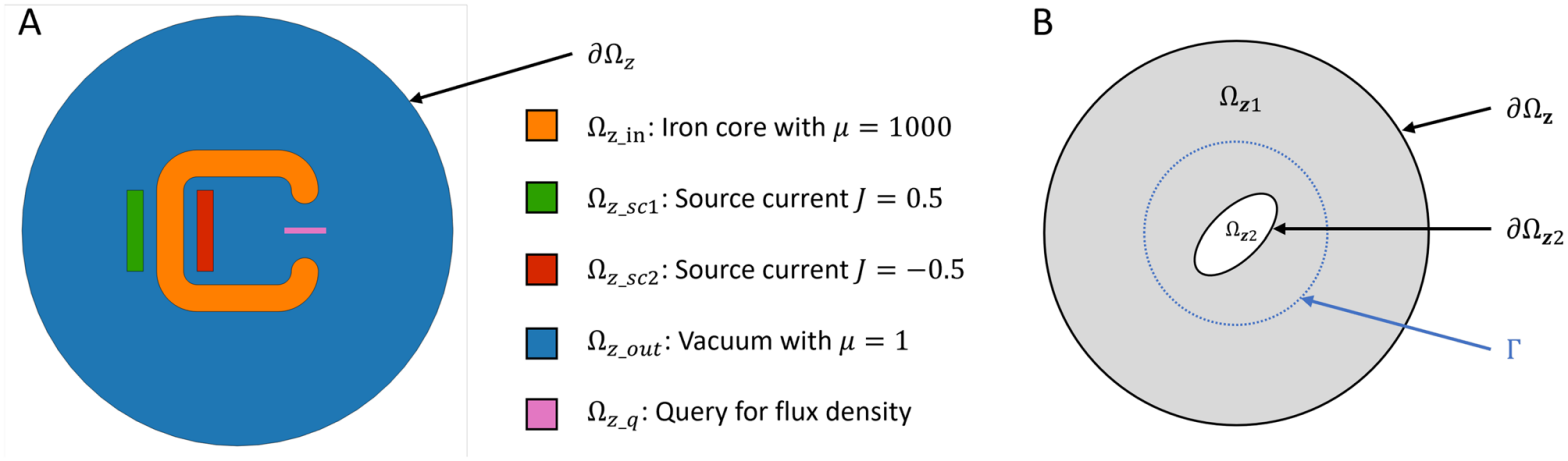
Reference domain shapes of two iron core shape optimization case studies: optimize for target magnetic flux density under current sources (**A**), and optimize for target electromagnetic torque subject to a uniform magnetic flux density boundary condition (**B**).

This work aims to address the discontinuity of material property fields in the context of physics-informed design optimization problems. Current solutions to this problem diverge into two paths: The first involves direct parameterization of material properties over the computation domain, such as density-based methods or level-set methods as detailed in^42,43^. To retain differentiability, the abrupt material property changes across subdomain interfaces are typically approximated through smooth transition functions. These approximations, while offering broad adaptability, might yield inaccurate results when subdomains have highly contrasting property values. The second path involves domain decomposition^44^. Exact material properties are assigned to collocation points within each decomposed subdomain while the PDE residual loss is replaced with a boundary condition loss on subdomain interfacial points, allowing more accurate PINN solutions to PDEs (an example shown in SI Appendix). While this ensures precise solutions regardless of property disparities across subdomain interfaces, the material properties on collocation points become non-differentiable as they are determined by indicator functions (if in a specific subdomain). In this case, an alternate shape parameterization method is essential. In this work, we propose to parameterize the property field design space through coordinate projection, in the form of a neural network. The proposed method decouples the definition of shapes and material properties, allowing exact material property representation through fixed spatially discontinuous functions, while providing differentiable parameterization of arbitrarily complex domain shapes. Moreover, the shape projection neural network can be smoothly incorporated with PINNs, enabling physics-informed shape optimization which offers significantly better adaptability towards practical engineering problems compared to classical methods. However, we want to make a note here that the current framework does not allow the change of topology, and should thus be treated as a shape optimization method. The proposed framework is showcased in optimizing the shape of iron cores in 2D static magnetic fields. The proposed optimization framework includes a domain decomposition PINN solver that can properly handle the large permeability contrast between neighboring subdomains. Therefore, the training process optimizes the shape and solves for the physics field simultaneously based on loss functions, without the aid of ground truth generated by external PDE solvers. The performance of the optimized iron core is subsequently validated by finite-element analysis (FEA) using commercial software COMSOL Multiphysics.

To showcase the capability of our proposed physics-informed shape optimization framework, we aim to optimize the shape of ferromagnetic iron cores for generating desired static magnetic fields. For simplicity, the two case studies are solved in 2D space with governing equations and neural network architectures described in the “Methods” section.

Figure 1A illustrates the reference domain shapes of a 2D C-shape iron core problem (the first case study). The reference iron core domain $\Omega _{z\_in}$ is initialized to be a C-shape with a thickness of 1 and relative permeability of 1000. When such an extreme property gap exists, PINN with domain decomposition provides more accurate solutions compared to domain smoothing (comparison given in SI Appendix). The iron core rests in a circular vacuum domain $\Omega _{z\_out}$ of radius 8 with two current sources $\Omega _{z\_sc1}$, $\Omega _{z\_sc2}$ of density 0.5 and $-\,0.5$ on its sides. The goal is to find a projection from $\Omega _{z\_in}$ to $\Omega _{x\_in}$ that generates some desired magnetic flux density within the query domain $\Omega _{z\_q}$.

Figure 1B illustrates the reference domain shapes of a magnetic torque problem (the second case study). The reference iron core domain $\Omega _{z2}$ is initialized to be an ellipse with major axis 1.5, minor axis 0.7, and $45^\circ$ inclination. The iron core rests in a circular vacuum domain $\Omega _{z1}$ of radius 8 with a uniform external magnetic flux density on boundary $\partial \Omega _z$. The existence of the iron core may distort the external magnetic field, yielding a magnetic torque that can be estimated by performing the integral in Eq. (11) along any close trajectory $\Gamma$ around the iron core. In this scenario, we assume infinite permeability on the iron core to examine a slightly different physics loss formulation (detailed in *Results and Discussion*), which yields similar solutions to any large relative permeability (e.g. $\mu =1000$).

**Figure 2:**
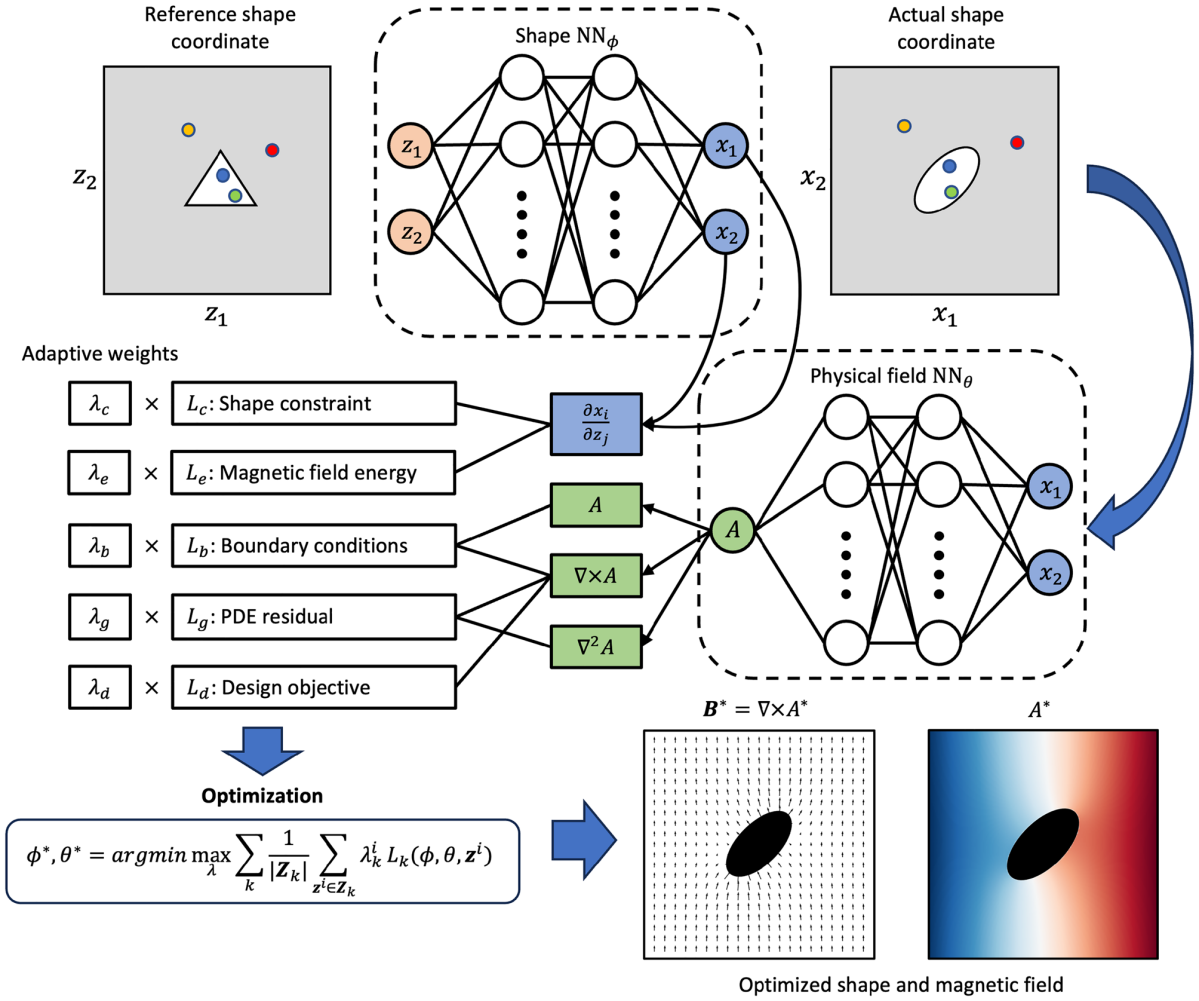
General framework for shape optimization using coordinate projection PINN. The shape network $NN_\phi$ projects the coordinates of the reference point cloud to the actual shape with topology preserved. In other words, the white ellipse on the projected domain $\Omega _x$ is represented by the same set of points sampled within the white triangle on $\Omega _z$, but projected by $NN_\phi$. The PINN $NN_\theta$ predicts physical fields on the actual shape coordinates. $\phi$ and $\theta$ are updated to minimize a loss function which is a combination of design objectives, shape constraints, and governing equations.

Figure 2 illustrates the entire optimization framework that will be implemented to address the case studies aforementioned; it consists of a shape neural network $NN_\phi$ and a physical field neural network $NN_\theta$ (formal definitions in the “Methods” section). In this work, shapes are defined using a reference shape and the shape neural network $NN_\phi$. The reference shape $\Omega _z$ is mathematically approximated by a point cloud with fixed coordinates ${\textbf{z}}$. The coordinates of the reference point cloud are projected by the shape neural network $NN_\phi$ to new coordinates ${\textbf{x}}$. This projected point cloud represents the optimized (deformed) shape once the neural networks are trained. Meanwhile, the positive Jacobian constraint is required for the entire point cloud to preserve topology and avoid any unphysical deformation. A typical PINN $NN_\theta$ is then responsible for predicting the correct physical field, specifically the magnetic vector potential (MVP) field in this work, over the projected spatial coordinates ${\textbf{x}}$. Such parameterization incorporates all design information within a neural network $NN_\phi$. As a result, it allows physics-informed loss functions defined on decomposed computation domains, while keeping all geometry features differentiable, including domain, boundary, and interface shapes. It is worth noting that defining shapes through boundary parameterization is not advisable. While it’s feasible to parameterize a smooth and closed curve, the material properties at domain collocation points become nondifferentiable. This is because they are determined by an indicator function determining whether they lie inside the curve.

The proposed physics-informed shape optimization framework is completely self-contained, learning physics and searching for better designs all by itself. Therefore, the loss function is composed of multiple components including residuals from strong and weak form governing equations, boundary conditions, design constraints, and design objectives, whose expressions depend on the actual problem of interest. The training process employs self-adaptive weights to effectively balance the contributions of loss functions from different sources. Each loss term is prefixed with adaptive weights $\lambda$. These weights are dynamically updated to maximize the overall loss, thereby placing greater emphasis on constraints that are not well met^45^. This allows user-defined design constraints to be added effortlessly as penalty functions, without derivation of Lagrangian multipliers^46^.

## Results

### Case study one

To optimize the C-shape iron core in case study one, the MVP field neural network $NN_\theta$ and $NN_\phi$ are initialized according to the “Methods” section. The training function (Eq. 1) is then calculated on point sets sampled from the given reference domains ${\textbf{Z}}_e, {\textbf{Z}}_g, {\textbf{Z}}_{c1}\subset \Omega _z$, ${\textbf{Z}}_{c2}\subset \Omega _{z\_in}$, ${\textbf{Z}}_b, {\textbf{Z}}_{c3}\subset \partial \Omega _z$, ${\textbf{Z}}_{c4}\subset \Omega _{z\_sc1}\cup \Omega _{z\_sc2}\cup \Omega _{z\_q}$, and ${\textbf{Z}}_d\subset \Omega _{z\_q}$.

1 $$\begin{aligned} \left\{ \begin{aligned}&L_g = |\nabla _{\textbf{x}}^2(NN_\theta \circ NN_\phi )({\textbf{z}})+\mu ({\textbf{z}})J({\textbf{z}})|^2 \quad \forall {\textbf{z}} \in {\textbf{Z}}_g \\&L_e = \frac{1}{|{\textbf{Z}}_e|}\sum _{{\textbf{z}}\in {\textbf{Z}}_e}\bigg (\frac{1}{2\mu ({\textbf{z}})}|\nabla _{\textbf{x}}\times NN_\theta \circ NN_\phi ({\textbf{z}})|^2-J({\textbf{z}})NN_\theta \circ NN_\phi ({\textbf{z}}))\cdot Jac_\phi ({\textbf{z}}) \\&L_b = |NN_\theta \circ NN_\phi ({\textbf{z}})|^2 \quad \forall {\textbf{z}} \in {\textbf{Z}}_b\\&L_{c1} = |ReLU(Jac_\phi ({\textbf{z}})-1.6)+ReLU(0.4-Jac_\phi ({\textbf{z}}))|^2 \quad \forall {\textbf{z}} \in {\textbf{Z}}_{c1} \\&L_{c2} = |\frac{1}{|{\textbf{Z}}_{c2}|}\sum _{{\textbf{z}}\in {\textbf{Z}}_{c2}} Jac_\phi ({\textbf{z}})-1|^2 \\&L_{c3} = |NN_\phi ({\textbf{z}})-{\textbf{z}}|^2 \quad \forall {\textbf{z}} \in {\textbf{Z}}_{c3} \\&L_{c4} = |NN_\phi ({\textbf{z}})-{\textbf{z}}|^2 \quad \forall {\textbf{z}} \in {\textbf{Z}}_{c4} \\&L_d = \left| \left( \frac{1}{|{\textbf{Z}}_d|}\sum _{{\textbf{z}}\in {\textbf{Z}}_d}\nabla _{\textbf{x}}\times NN_\theta \circ NN_\phi ({\textbf{z}})\cdot [0,1]^T \right) -B_{target}\right| ^2=|B_q-B_{target}|^2 \\&L = \lambda _eL_e+\lambda _dL_d+\lambda _{c2}L_{c2}+\sum _{k\in \{c1,c3,c4,b,g\}}\frac{1}{|{\textbf{Z}}_k|}\sum _{{\textbf{z}}^i\in {\textbf{Z}}_k}\lambda _k^i L_k(\phi ,\theta ,{\textbf{z}}^i) \end{aligned} \right. \end{aligned}$$

**Figure 3:**
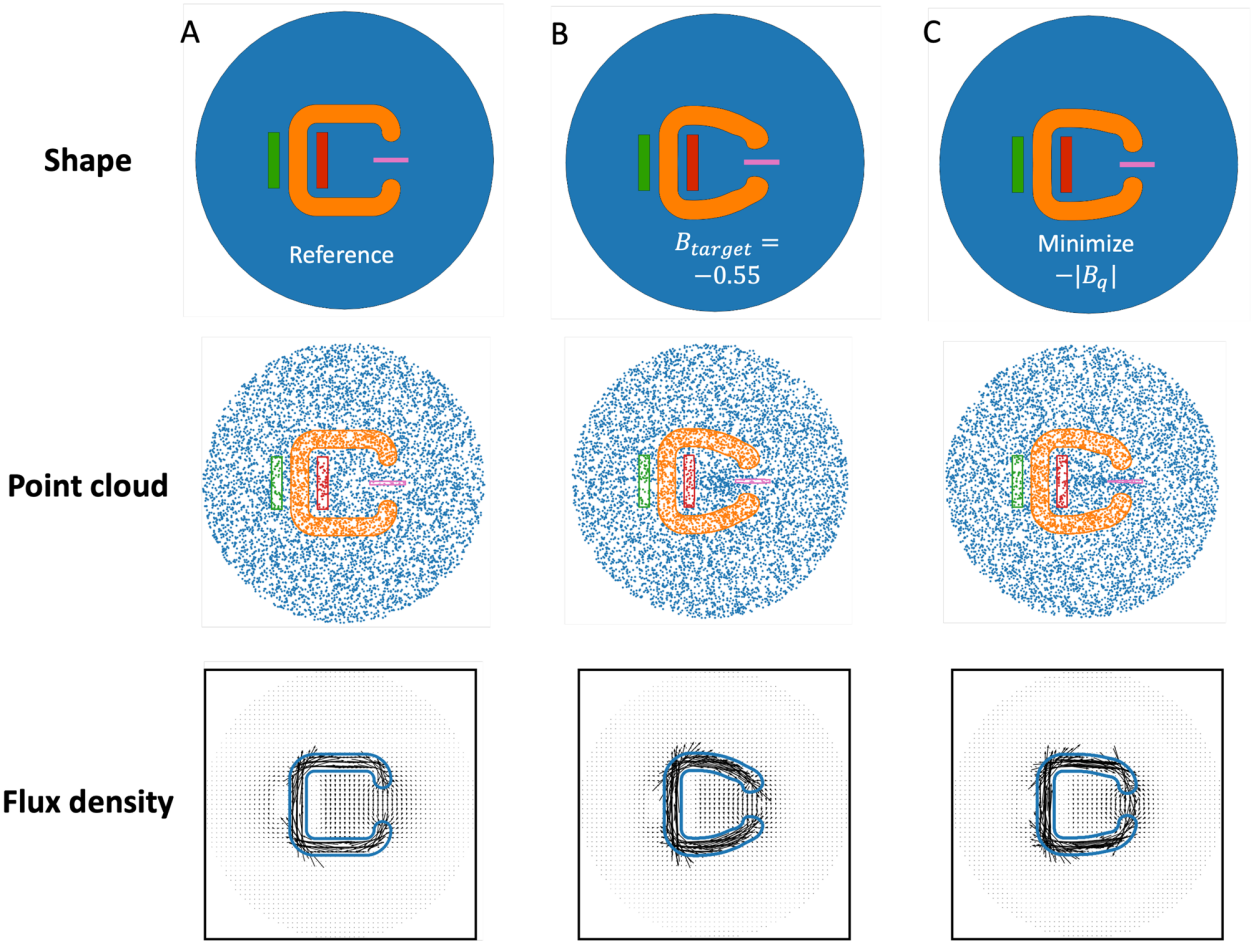
Comparison of domain shapes and magnetic flux density fields among the reference (**A**), the optimized iron core for $B_{target}=-\,0.55$ (**B**), and the optimized iron core for maximizing magnetic flux density (**C**). The second row shows a point cloud approximation of the reference shape and the optimized shapes. Boundaries are highlighted to help distinguish different subdomains. For better visualization, the plots only show $10\%$ of the actual point cloud used for training.

$NN_\theta \circ NN_\phi$ represents the composition of the shape and the physics neural networks, projecting a reference coordinate ${\textbf{z}}$ to the predicted MVP value on the corresponding spatial coordinate ${\textbf{x}}$. $L_b$ in Eq. (1) calculates the strong form PDE residual (Eq. 6) for each collocation point, which is the most commonly seen domain loss in PINNs. $L_e$ facilitates a Monte Carlo estimation of the magnetic energy (Eq. 7). A Jacobian factor is multiplied to $L_e$ as the collocation points are no longer uniformly distributed on the projected spatial coordinate ${\textbf{x}}$. A valid MVP solution should minimize this energy loss. Notice that minimizing the strong form $L_g$ or the weak form $L_e$ would produce the same MVP field solution. However, incorporating both forms into the final loss function proves to be advantageous in navigating local minima, especially when seeking a continuous MVP solution on a heavily discontinuous permeability field (more details discussed in SI Appendix). The Dirichlet boundary condition is addressed in $L_b$ which penalizes any non-zero boundary MVP. $L_{c1}$ preserves topology by constraining Jacobian within a certain positive range to avoid infeasible or highly distorted shape changes. $L_{c2}$ penalizes volume change in the iron core. $L_{c3}$ and $L_{c4}$ prohibit deformation at the outer boundary, current sources, and the query region as they are fixed external objects that are not part of the design variables. $L_d$ is the objective function with a target value for the vertical component of the magnetic flux density in the query domain. To best satisfy the design goal, any $B_q$ values that deviate from $B_{target}$ will be penalized. The total training loss *L* is a weighted summation of the abovementioned loss components. Self-adaptive updating is utilized to automatically adjust the loss weights except for $\lambda _e$ which has a fixed value of 3.3. This is due to the fact that the minimal value of magnetic energy $L_e$ isn’t zero. Meanwhile, we notice that all loss terms should ideally stay at 0 values regardless of the projected shape, except for $L_e$ which should only be minimized given a fixed $NN_\phi$. Therefore, $\frac{\partial L_e}{\partial \phi }$ is excluded from the computation graph so that the energy minimization loss $L_e$ only affects the physics model $NN_\theta$ without directly deforming $NN_\phi$.

To calculate the total training loss, 35,600 random collocation points are sampled over the entire domain $\Omega _{z}$ and shared by ${\textbf{Z}}_e$, ${\textbf{Z}}_g, {\textbf{Z}}_{c1}$. Another 5000 random collocation points are sampled in the reference iron core domain for ${\textbf{Z}}_{c2}$. 6000 uniform boundary points are sampled for ${\textbf{Z}}_{c3}$. 300, 300, and 66 points are sampled for ${\textbf{Z}}_{sc1}$, ${\textbf{Z}}_{sc2}$ and ${\textbf{Z}}_q$ to constrain shape change. The sampled training points together with the neural networks (architectures detailed in the “Methods” section) take approximately 4 GB of GPU memory. $NN_\phi$ is first initialized to make identity prediction ${\textbf{z}}=NN_\phi ({\textbf{z}})$ over the entire domain $\Omega _z$ through 8000 epochs of supervised training. At this initialization stage, the ground truth label is identical to the input coordinate ${\textbf{z}}$. $NN_\phi$ and $NN_\theta$ are then updated simultaneously by minimizing the complete loss function *L* in Eq. (1). Initial learning rates are set as 0.001 for $\phi$ and 0.002 for $\theta$, where both decay exponentially by a factor of 0.9 for every 1000 epochs, with a total of 60,000 epochs.

**Figure 4:**
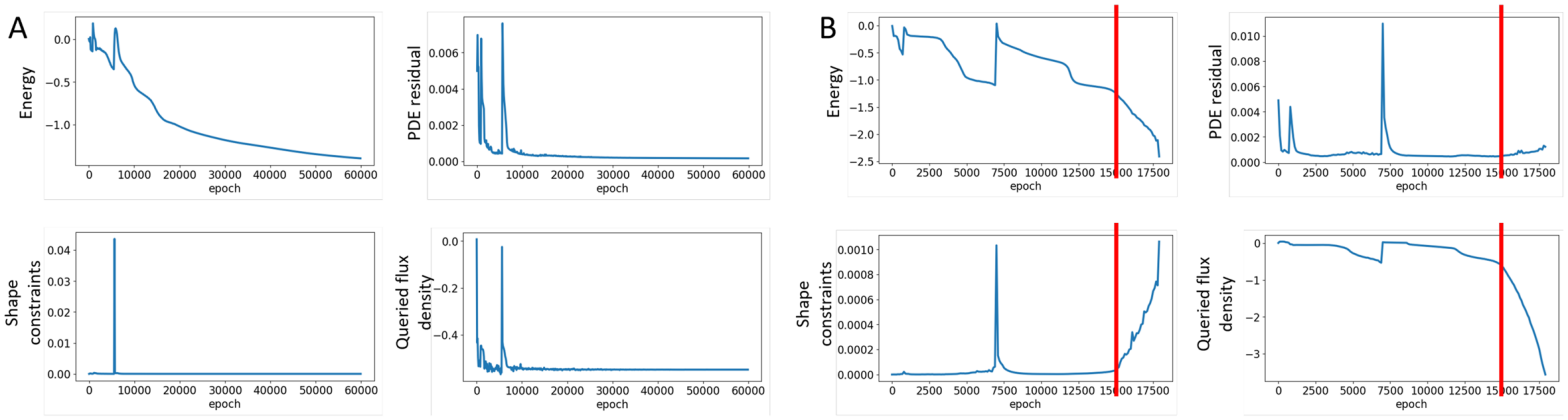
Evolution of magnetic energy, PDE residual, shape constraint losses, and the queried vertical magnetic flux density $B_q$ over the training process for $NN_\theta$ and $NN_\phi$, when optimizing for a target flux density $B_{target}=-\,0.55$ (**A**) and directly minimizing $-|B_q|$ (**B**). The red lines highlight the model checkpoint.

We first solve the magnetic flux density field ${\textbf{B}}$ for the initial reference C-shape iron core by holding $NN_\phi$ to be the constant identity mapping. The solution is shown in Fig. 3A with a vertical flux density of $B_q=-\,0.34$ at the query region. A similar value of $B_q=-\,0.36$ is computed by FEA with COMSOL, validating the formulation of the physics loss. Figure 3B shows the optimized iron core shape projected by the trained $NN_\phi$ when the design objective is set as $B_{target}=-0.55$. It can be observed that the training process attempts to pull the iron core towards the query region to enhance the magnetic flux around the query domain. The training curves are plotted in Fig. 4A, including the evolution of magnetic energy, governing equation (PDE) residual $L_g$, shape constraint losses $L_{c1}-L_{c4}$, and the queried vertical magnetic flux density. We notice that all zero target constraints (including $L_d$) in the training curves converge relatively fast within 10,000 epochs, whereas the remaining training process focuses on correctly resolving the physical fields by minimizing the magnetic energy. The optimized iron core contour is exported and validated in COMSOL, providing $B_q=-\,0.491$. The difference between the queried flux density from PINN ($B_q=-\,0.55$) and COMSOL ($B_q=-\,0.491$) is likely caused by two major sources: numerical discrepancy between Monte Carlo sampling and shape function approximation (FEA), and balance among multiple penalty losses over training.

We also explored optimizing the iron core shape by switching the design objective ($L_d$ in Eq. 1) to $L_d=-\,|B_q|$, aiming to directly reduce the vertical flux density within the query area. In this case, the objective function $L_d$ lacks a zero minimum. As a result, a fixed value 0.005 is assigned to $\lambda _d$, with adaptive weight update disabled. Minimizing $B_q$ without a target value makes the problem more challenging as it permits the violation of physical and shape constraints, especially with extreme $B_q$ values. To avoid exhaustive hyperparameter searching, we choose to record the training progression at every 500 epochs, subsequently selecting a suitable checkpoint model based on the observed training trends.

Figure 4B plots the training progress during the optimization of the iron core to achieve the minimum value of $-|B_q|$. We select the model from epoch 15,000 as the checkpoint, given that it manifests the lowest energy and $B_q$ values before the shape constraints and PDE residual begin to evolve sharply. Post the 15,000 epoch mark, the shape projection model, denoted as $NN_\phi$, appears to either inflate the volume of the iron core or induce unphysical shape changes (negative Jacobian). This leads to hallucinated readings for magnetic energy and flux density. It is worth noting that the direct minimization of $-|B_q|$ enables the two neural networks to adapt more rapidly compared to the approach where a target value, $|B_q-B{target}|^2$, is specified. This acceleration is mainly attributed to the small constant weight of 0.005 associated with the direct minimization of objective function. In contrast, when a specific target value is present, the use of initially randomized adaptive weights takes additional epochs to rectify the physical field prediction. However, the application of adaptive weights in front of a zero-target loss can greatly alleviate the efforts required for hyperparameter tuning.

In Fig. 3C, we present the iron core’s optimized shape and the corresponding predicted magnetic flux density. The deformation observed here is similar to that in Fig. 3B, but with the core tips drawn more proximate to the query region. Specifically, while Fig. 3B shows a tendency to “bend” the core tips towards the query area, Fig. 3C seems to “extend” the tips by eliminating material from other regions. Parameterizing the shape change through a coordinate projection neural network brings huge freedom to the design space and yields infinite solutions, which depend both on the form of objective function and hyperparameters, especially fixed weights $\lambda _e$ and $\lambda _d$. The projected iron core contour is exported and validated in COMSOL, giving $B_q=-\,0.561$, a stronger magnetic flux than the previous targeted optimization. However, the symmetric form of $NN_\phi$ as outlined in Eq. (12), coupled with the penalty on positive Jacobian, prevents the algorithm from extending the iron core further towards the query domain. It is not surprising that the shape projection PINN overestimates the design objective ($B_q$ is approximately $-\,0.7$ from Fig. 4B), primarily owing to the involvement of multiple penalty constraints.

### Case study two

The electromagnetic torque generated by an iron core subject to a uniform magnetic flux density boundary condition (illustrated in Fig. 1B) can be calculated by Eqs. (10) and (11) in the “Methods” section based on the MVP field solution $NN_\theta$. Therefore, to find a proper iron core shape $NN_\phi$ that generates some target torque, we minimize the following training function (Eq. 2) that is calculated on point sets sampled from the reference domains ${\textbf{Z}}_g,{\textbf{Z}}_{c1}, {\textbf{Z}}_{c2}\subset \Omega _{{\textbf{z}}1}$, ${\textbf{Z}}_{b1},{\textbf{Z}}_{c3}\subset \partial \Omega _{\textbf{z}}$, ${\textbf{Z}}_{b2},{\textbf{Z}}_{c4}\subset \partial \Omega _{{\textbf{z}}2}$, and ${\textbf{X}}_d\subset \Gamma$:

**Figure 5:**
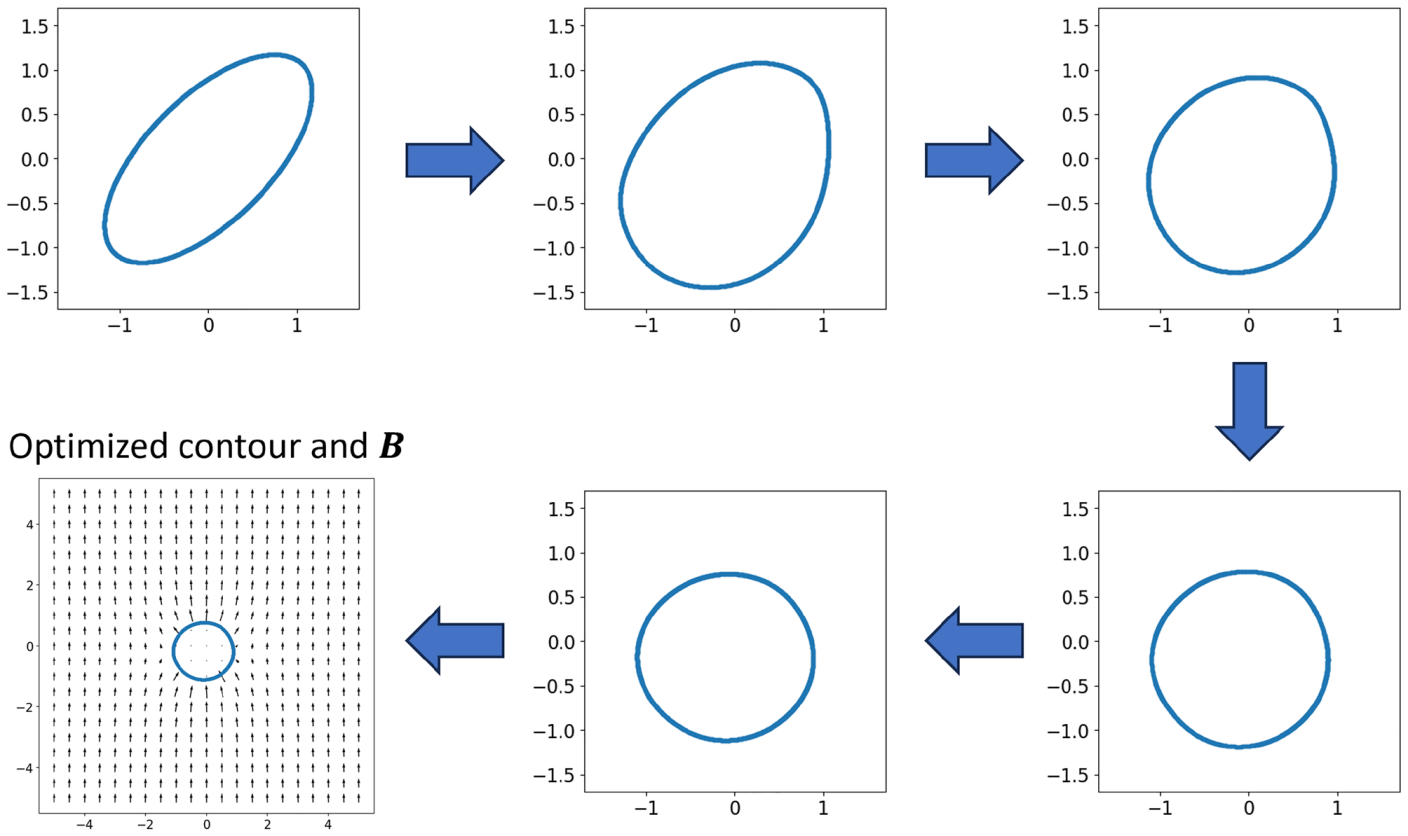
Evolution of the projected iron core contour over training, when target torque is set to 0. The final optimized contour shape and flux density field are shown on the bottom left.

2 $$\begin{aligned} \left\{ \begin{aligned}&L_g = |\nabla _{\textbf{x}}^2(NN_\theta \circ NN_\phi )({\textbf{z}})+\mu ({\textbf{z}})J({\textbf{z}})|^2 \quad \forall {\textbf{z}} \in {\textbf{Z}}_g \\&L_{b1} = |\nabla _{\textbf{x}}\times NN_\theta \circ NN_\phi ({\textbf{z}})-[0,1]^T|^2 \quad \forall {\textbf{z}} \in {\textbf{Z}}_{b1} \\&L_{b2} = |\nabla _{\textbf{x}}\times NN_\theta \circ NN_\phi ({\textbf{z}})\cdot {\textbf{t}}|^2 \quad \forall {\textbf{z}} \in {\textbf{Z}}_{b2} \\&L_{c1} = |ReLU(Jac_\phi ({\textbf{z}})-1.6)+ReLU(0.4-Jac_\phi ({\textbf{z}}))|^2 \quad \forall {\textbf{z}} \in {\textbf{Z}}_{c1} \\&L_{c2} = |\frac{1}{|{\textbf{Z}}_{c2}|}\sum _{{\textbf{z}}\in {\textbf{Z}}_{c2}} Jac_\phi ({\textbf{z}})-1|^2 \\&L_{c3} = |NN_\phi ({\textbf{z}})-{\textbf{z}}|^2 \quad \forall {\textbf{z}} \in {\textbf{Z}}_{c3} \\&L_{c4} = |ReLU(curv(NN_\phi ({\textbf{z}}))-5)|^2 \quad \forall {\textbf{z}} \in {\textbf{Z}}_{c4} \\&L_d = |\sum _{{\textbf{x}}\in {\textbf{X}}_d}{\textbf{r}}({\textbf{x}})\times ({\textbf{T}}({\textbf{x}})\cdot {\textbf{n}}({\textbf{x}}))d-\tau _{target}|^2 \\&L = \lambda _dL_d+\lambda _{c2}L_{c2}+\sum _{k\in \{c1,c3,c4,b1,b2,g\}}\frac{1}{|{\textbf{Z}}_k|}\sum _{{\textbf{z}}^i\in {\textbf{Z}}_k}\lambda _k^i L_k(\phi ,\theta ,{\textbf{z}}^i) \end{aligned} \right. \end{aligned}$$

The governing equation residual loss $L_g$ remains the same as in Eq. (1). As we are assuming infinite permeability over the iron core domain $\Omega _{{\textbf{z}}2}$ (Fig. 1B), both $NN_\phi$ and $NN_\theta$ are defined only in $\Omega _{{\textbf{z}}1}$. Therefore, Neumann boundary conditions (Eqs. 8 and 9) are needed on $\partial \Omega _{{\textbf{z}}2}$ to correctly solve the MVP field. Although the magnetic flux density ${\textbf{B}}$ isn’t properly defined in a domain with infinite permeability, the tangential component of magnetic field strength ${\textbf{H}}$ should always be 0 due to the infinite denominator as implemented in Eq. (2) $L_{b1}$. Meanwhile, the energy loss $L_e$ is no longer necessary as the entire computation domain is homogeneous. $L_{c1}$ is again added to penalize any unphysical deformation, while $L_{c2}$ conserves the total volume. $L_{c3}$ holds still the external boundary of the computation domain so that only the iron core is deformed. $L_{c4}$ penalizes any large curvature on $\partial \Omega _{{\textbf{z}}2}$ that is beyond 5. The design objective function $L_d$ computes the squared distance between the target torque and the magnetic torque which is numerically estimated on $\Gamma$. The total training loss *L* is again a weighted summation of all the loss components in Eq. (2) through the self-adaptive training scheme.

To calculate the total training loss, 30,000 random collocation points are sampled within the vacuum domain $\Omega _{z1}$ and shared by ${\textbf{Z}}_g,{\textbf{Z}}_{c1}, {\textbf{Z}}_{c2}$. 6000 uniform boundary points are sampled and shared by ${\textbf{Z}}_{b1}, {\textbf{Z}}_{c3}$. 1250 uniform boundary points are sampled and shared by ${\textbf{Z}}_{b2}, {\textbf{Z}}_{c4}$. A set of 800 equally spaced query points $X_d$ is sampled along $\Gamma$ (a circle of radius 4, centered at the origin) to estimate $\tau$. Notice that $X_d$ (and $\Gamma$) is defined on the projected space ${\textbf{x}}$ instead of the reference space ${\textbf{z}}$ to avoid unnecessary design parameters. $NN_\phi$ is first initialized to make identity prediction ${\textbf{z}}=NN_\phi ({\textbf{z}}), \forall {\textbf{z}}\in {\textbf{Z}}_g$ through 8000 epochs of supervised training. $NN_\phi$ and $NN_\theta$ are then updated simultaneously by minimizing the complete loss function *L* in Eq. (2). Initial learning rates are set as 0.0005 for $\phi$ and 0.005 for $\theta$, where both decay exponentially by a factor of 0.9 for every 1000 epochs, with a total of 28000 epochs.

Figure 5 shows the evolution of $\partial \Omega _{{\textbf{z}}2}$ projected by $NN_\phi$ over the training procedure, with a zero target torque $\tau _{target}=0$. The pronounced permeability disparity between the iron core and the vacuum causes the external boundary’s uniform magnetic flux density ${\textbf{B}}=[0,1]^T$ to distort. This distortion results in the MVP field exerting a torque on the initially inclined elliptical iron core. When estimated using FEA simulation and assuming a permeability gap multiplied by 1000, this torque amounts to 2.849. The zero torque optimization problem technically has infinitely many solutions, including any ellipses whose main or minor axis is aligned with the external magnetic flux density. It is observed that the training process eventually converges to the circular shape as shown on the bottom left of Fig. 5. This shape seems to be the optimization algorithm’s preference for any random seed. The magnetic flux density inside of the iron core is not well-defined and is thus masked from all the plots. The final contour (projected $X_d$) is exported and verified with FEA, with iron core permeability set to 1000. According to FEA result, the optimized iron core produces a minuscule torque of 0.017, a value substantially smaller than the original torque. It’s worth noticing that our shape projection parameterization method offers a versatile way to incorporate design constraints, like the curvature penalty in Eq. (2). However, it does present challenges in converging precisely to an optimal solution, such as a perfect circle.

**Figure 6:**
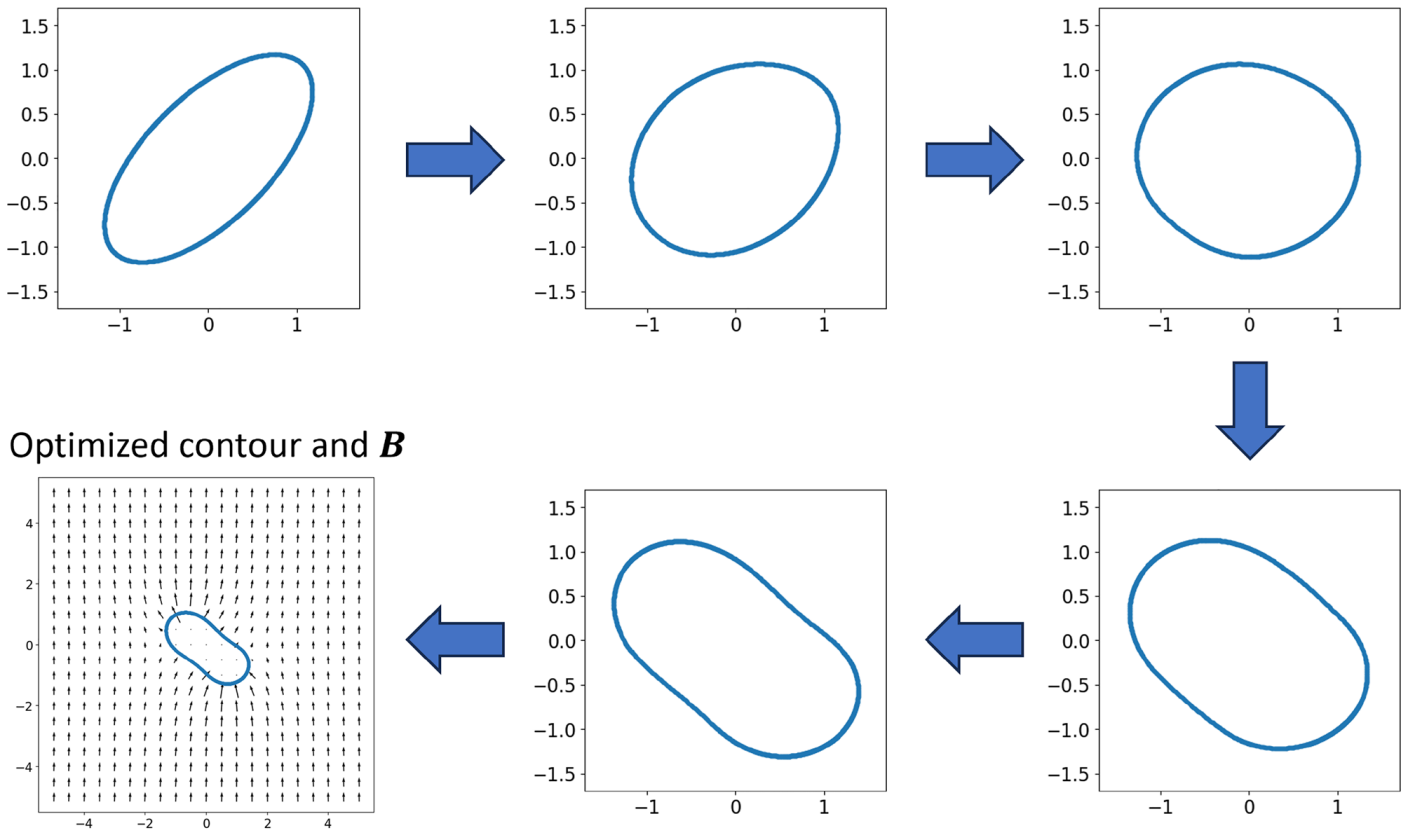
Evolution of the projected iron core contour over training, when target torque is set to $-\,3$. The final optimized contour shape and flux density field are shown on the bottom left.

Figure 6 shows the evolution of $\partial \Omega _{{\textbf{z}}2}$ projected by $NN_\phi$ over the training procedure, under a different scenario with a target torque of $\tau _{target}=-3$. As the original shape generates a torque of 2.849, we expect the final optimized shape to be similar to the reflection of the original ellipse about the vertical axis. The training process eventually converges to the shape as shown on the bottom left of Fig. 6, with a peanut-like shape inclined to the left. Owing to the application of Jacobian penalty $L_{c1}$ and curvature penalty $L_{c4}$, a smooth shape transition can be observed where the iron core gets compressed gradually along its main axis and then extended to the opposite direction. The magnetic flux density inside of the iron core is again masked due to infinite permeability. The final contour is exported and verified in COMSOL, with iron core permeability set to 1000. The optimized iron core reports a torque of $-3.105$ from FEA simulation, agreeing well with the design objective.

## Discussion

The rapid advancement of AI has showcased its capabilities in tackling complex material design challenges. Among various methodologies, PINN is notable for its ability to perform self-supervised learning in physics problems. Several researchers have demonstrated the feasibility of optimizing material topology using physics-informed machine learning, showing great promise. Nonetheless, a potential obstacle for the broad adoption of PINN is its inherent need for continuity, particularly in scenarios with multiple domains having distinct properties. Oftentimes, this continuity is achieved by introducing smooth transition functions at domain boundaries. However, this approximation can lead to inaccuracies when the property difference is significant.

In this work, we addressed the field discontinuity challenge in physics-informed material design optimization problems by introducing the shape projection neural network $NN_\phi$. Unlike a direct shape definition through boundary curve parameterization, $NN_\phi$ parameterizes the shape in an implicit manner, thus requiring a point cloud to keep track of the reference shape. However, this approach is very beneficial in the context of physics-informed machine learning where the geometric features of all training points (including domain collocation points and boundary points) should be differentiable from an objective function. Once the reference point cloud is projected through $NN_\phi$, it can be used as training points to compute the residual loss of any physics field neural network $NN_\theta$ or a design objective. The cumulative loss function is then backpropagated to correct physics ($\theta$) and shape design ($\phi$) simultaneously.

The proposed framework is applied to optimize iron core designs in two benchmark magnetostatic problems: shape optimization of a C-shape iron core to generate a concentrated magnetic field in a query region subject to current sources, and shape optimization of an elliptical iron core to generate target electromagnetic torque subject to a uniform magnetic flux density boundary condition. FEA simulation is used to validate the performance of the optimized iron core designs. The following takeaways can be summarized from our results: The shape projection method offers robust expressiveness for parameterizing a wide range of shapes with both smooth and sharp features; Physics can be solved with domain decomposition, eliminating the need for transition function or intermediate material properties; This framework is capable of solving physics and optimizing domain shapes simultaneously, operating entirely without the need for external data; The training process is efficient (both case studies take approximately 1 hour to train) and accurate (validation result shows a small discrepancy from the target value); Classical optimization techniques (either density-based or level-set parameterizations) require strict derivation of design sensitivity which is tedious or sometimes intractable, while adding custom constraints and design objectives is straightforward (as seen in Eqs. 1 and 2) through the proposed framework by incorporating penalty functions directly into the self-adaptive training loss. On the other hand, the following perspectives should be further studied or improved in the future: The current framework only allows shape optimization on a fixed topology; The involvement of constraints as penalty loss functions makes the optimization process difficult to converge precisely at the target objective value; The shape neural network tends to learn a projection that’s in the vicinity of the reference domain shape, emphasizing the importance of shape initialization (reference point cloud). Future work can be dedicated to studying the effect of reference shape, and domain reinitialization to address unsatisfactory reference shapes. Besides, a more comprehensive investigation is needed to understand how various hyperparameters (such as model architectures, weight initialization, learning rate, optimization scheduler, etc.) affect the training performance, especially the density of collocation points which dominates the accuracy of shape and physics approximations.

## Methods

### Governing physics equations

The governing PDEs for a magnetostatic problem in 2D space can be expressed in the following general forms:

3 $$\begin{aligned}{} & {} {\textbf{B}} = \nabla _{\textbf{x}}\times {\textbf{A}} = \nabla _{\textbf{x}}\times (A{\textbf{e}}_3) \end{aligned}$$

4 $$\begin{aligned}{} & {} \nabla _{\textbf{x}}\times {\textbf{H}} = {\textbf{J}}=(J{\textbf{e}}_3) \end{aligned}$$

5 $$\begin{aligned}{} & {} {\textbf{B}} = \mu {\textbf{H}} \end{aligned}$$

where *A* is the out-of-plane component (${\textbf{e}}_3$) of the magnetic vector potential (MVP) field which is treated as a scalar field in the 2D plane and ${\textbf{B}}$ is the magnetic flux density vector. The introduction of MVP field automatically satisfies Gauss’s law requiring the divergence of ${\textbf{B}}$ to be always 0^47^. ${\textbf{H}}$ is the magnetic field strength vector, $\mu$ is the magnetic permeability, *J* is the scalar value current density (perpendicular to the 2D plane), and the subscript ${\textbf{x}}$ of the curl operator indicates the corresponding coordinate system that spatial differentiation is taken. Proper Dirichlet or Neumann boundary conditions on $\partial \Omega$ are needed for a unique solution of *A* or ${\textbf{B}}$. When permeability is a constant locally, Eqs. (3–5) can be rewritten more compactly as:

6 $$\begin{aligned} \nabla _{\textbf{x}}^2 A = -\mu J \end{aligned}$$

One can alternatively obtain the MVP solution to a 2D magnetostatic problem by minimizing the magnetic energy $E_B$ defined by

7 $$\begin{aligned} E_B = \int _\Omega (\frac{1}{2\mu }|{\textbf{B}}|^2-JA) d\Omega . \end{aligned}$$

Evaluating $E_B$ requires the magnetic field over the entire computation domain $\Omega$. $E_B$ is proven to a minimum when the weak form of Eqs. (3–5) is solved^48^.

When domain decomposition is needed, one can use Divergence and Green’s Theorem to rewrite Eqs. (3) and (4) as Neumann boundary conditions on the domain boundary (interface):

8 $$\begin{aligned}{} & {} {\textbf{B}}_{1}\cdot {\textbf{n}}={\textbf{B}}_{2}\cdot {\textbf{n}} \end{aligned}$$

9 $$\begin{aligned}{} & {} {\textbf{H}}_{1}\cdot {\textbf{t}}={\textbf{H}}_{2}\cdot {\textbf{t}} \end{aligned}$$

where the normal component of ${\textbf{B}}$ and the tangential component of ${\textbf{H}}$ should always remain continuous across any material boundary; the subscript indicates the region where the fields are evaluated. Note that all physical quantities are dimensionless in this work.

Given the magnetic flux density field ${\textbf{B}}$, one can further calculate the magnetic stress tensor ${\textbf{T}}$ and magnetic torque $\tau$:

10 $$\begin{aligned}{} & {} {\textbf{T}}=\frac{1}{\mu }\left( {\textbf{B}}{\textbf{B}}^T-\frac{1}{2}{\textbf{I}}|{\textbf{B}}|^2 \right) \end{aligned}$$

11 $$\begin{aligned}{} & {} \tau =\int _\Gamma {\textbf{r}}\times (\mathbf {T{\textbf{n}}})dS \end{aligned}$$

where ${\textbf{I}}$, ${\textbf{r}}$, ${\textbf{n}}$, and $\Gamma$ denote the identity matrix, position vector, normal vector, and some integration trajectory.

### Neural network architectures

In this work, we use neural networks to define the MVP field as $NN_\theta : \Omega _{\textbf{x}}\rightarrow R$ and the shape projection parameterization as $NN_\phi : \Omega _{\textbf{z}}\rightarrow \Omega _{\textbf{x}}$. $NN_\theta$ takes the spatial coordinate ${\textbf{x}}\in \Omega _{\textbf{x}}$ as input and predicts *A*. As the MVP field is always continuous over the space regardless of material properties, it is more suitable to be represented as neural networks. For the conciseness of mathematical expressions, the predicted vector form MVP field $NN_\theta ({\textbf{x}}){\textbf{e}}_3$ is abbreviated as $NN_\theta ({\textbf{x}})$ in this work. $NN_\phi$ takes the material coordinate ${\textbf{z}}\in \Omega _{\textbf{z}}$ as input and predicts the corresponding spatial coordinate. This projection helps define the actual optimized shape $\Omega _{\textbf{x}}$ projected from a given reference shape $\Omega _{\textbf{z}}$ through $\phi$. In the first case study, the shape projection is defined to be an odd function in the vertical coordinate to enforce symmetry:

12 $$\begin{aligned} NN_\phi ({\textbf{z}})=[z_1,z_2]^T&+[1,1]^T\cdot {\hat{NN}}_\phi (z_1,z_2) +[1,-1]^T\cdot {\hat{NN}}_\phi (z_1,-z_2) \end{aligned}$$

While in the second case study, the shape function is simply:

13 $$\begin{aligned} NN_\phi ({\textbf{z}})=[z_1,z_2]^T+{\hat{NN}}_\phi ({\textbf{z}}) \end{aligned}$$

Both $NN_\theta$ and ${\hat{NN}}_\phi$ share the same architecture with 6 hidden layers of width 50 and the hyperbolic tangent activation. Note that the network architecture is directly adapted from^31^ without hyperparameter tuning, as the focus of this work is on the general physics-informed shape optimization framework. The training of $NN_\phi$ and $NN_\theta$ is conducted using Pytorch and DeepXDE on an NVIDIA A40 GPU. Training point sampling, loss functions, and optimization strategies are detailed in the corresponding sections.

### Supplementary Information

Supplementary Information.

## Data Availability

The datasets generated and analysed during the current study are available from the corresponding author on reasonable request.
