# Supplementary material for: Physics-informed shape optimization using coordinate projection
Source: Sci Rep. 2024 Mar 19;14:6537. doi: 10.1038/s41598-024-57137-4 (PMC10951326; doi:10.1038/s41598-024-57137-4)
Supplement: Supplementary file 1 — Supplementary Information. [file 41598_2024_57137_MOESM1_ESM.pdf]

Supplementary Information for: Physics-informed shape optimization using coordinate projection

Zhizhou Zhang[a], Chungwei Lin[a], and Bingnan Wang[a*]

[a]Mitsubishi Electric Research Laboratories, 201 Broadway, 8th Floor, Cambridge, MA 02139-1955, US
[*]Correspondence: bwang@merl.com

1 Computation domain with distinct permeability

In the main manuscript, we evaluate the proposed shape projection algorithm on two-dimensional (2D) static magnetic problems governed by the following equations[1]:

$$\mathbf{B} = \nabla_{\mathbf{x}} \times \mathbf{A} = \nabla_{\mathbf{x}} \times A\mathbf{e}_3 \tag{1}$$

$$\nabla_{\mathbf{x}} \times \frac{\mathbf{H}}{\mu} = \mathbf{J} = J\mathbf{e}_3 \tag{2}$$

where $\mathbf{A}$ is the magnetic vector potential (MVP) field and $\mathbf{B}$ is the magnetic flux density vector[1]. $\mathbf{H}$ is the magnetic field strength vector, μ is the magnetic permeability, $\mathbf{J}$ is the current density, and the subscript $\mathbf{x}$ of the curl operator indicates the corresponding coordinate system that spatial differentiation is taken. In 2D, $\mathbf{A}$ and $\mathbf{J}$ only have the out-of-plane component and are respectuively represented by $\mathbf{A} = A\mathbf{e}_3$, $\mathbf{J} = J\mathbf{e}_3$.

One would immediately notice that Eq. 2 requires taking the curl of $\frac{1}{\mu}$, thus requiring relative permeability to be everywhere continuous over the computation domain of a physics-informed neural network (PINN). However, for scenarios involving high-μ materials such as iron cores, the permeability contrast between neighboring environments can be easily larger than 1000. To address the challenges of such discontinuity, researchers have suggested two strategies: employing a smooth transition function to approximate the boundary between discontinuous properties, or training separate PINNs on each domain of same permeability and imposing the boundary conditions across interfaces.

Fig. S1 shows a simple computation domain consisting of two subdomains $\Omega = \Omega_1 \cup \Omega_2$ (sometimes more), representing the vacuum environment (a square domain with length 10) and an iron core (a circular domain center at the origin with radius 2). $\partial\Omega$ represents the outer boundary with a uniform magnetic flux density boundary condition, and $\partial\Omega_2$ defines the interface between material and vacuum. We will train PINNs to solve the MVP field of this simple problem to compare the performance of the two abovementioned strategies.

Domain property smoothing

Before solving the MVP field, the permeability should be smoothed across the material interface $\partial\Omega_2$. This is achieved by a Sigmoid transition function Eq. 3:

$$\mu(\mathbf{x}) = \frac{e^{-(r(\mathbf{x})-r_0)/k}}{1+e^{-(r(\mathbf{x})-r_0)/k}} \tag{3}$$

where r represents the distance from origin, r_0 denotes the radius of Ω_2, and k controls the level of smoothness, allowing Eq. 2 to be tractable by PINN on $\partial\Omega_2$. When the iron core has a relative permeability $\mu = 10$ while the vacuum has $\mu = 1$, fig. S2A illustrates the effect of Eq. 3 with a smoother transition at $k = 0.3$ and a steeper transition at $k = 0.03$.

The MVP field is then solved following the typical PINN framework[2]. A neural network $NN_\theta : \Omega \rightarrow R$ is defined to approximate the MVP field A, with 6 hidden layers of width 50 and hyperbolic tangent activation. For the conciseness of mathematical expressions, the predicted vector form MVP field $NN_\theta(\mathbf{x})\mathbf{e}_3$ is abbreviated as $NN_\theta(\mathbf{x})$

in this work. NN_θ is trained by penalizing the following loss functions through collocation point sets $\mathbf{X}_g$ uniformly sampled from Ω, and boundary point set $\mathbf{X}_b$ uniformly sampled from $\partial\Omega$:

$$\begin{cases} L_g = |\nabla_\mathbf{x} \times (\nabla_\mathbf{x} \times NN_\theta(\mathbf{x})/\mu(\mathbf{x})) - J(\mathbf{x})|^2 \quad \forall \mathbf{x} \in \mathbf{X}_g \\ L_b = |\nabla_\mathbf{x} \times NN_\theta(\mathbf{x}) - [0,1]^T|^2 \quad \forall \mathbf{x} \in \mathbf{X}_b \\ L = \sum_{k \in \{b,g\}} \frac{1}{|\mathbf{X}_k|} \sum_{\mathbf{x}^i \in \mathbf{X}_k} \lambda_k^i L_k(\theta, \mathbf{x}^i) \end{cases} \tag{4}$$

where L_g is the strong form residual loss corresponding to Eq. 1 and 2. L_b is the Dirichlet boundary loss with a uniform magnetic flux density $[0,1]^T$. The complete loss function L is a weighted sum of L_g and L_b. A self-adaptive training scheme[3] is utilized in this work to perform the min-max optimization as shown in Eq. 5. Self-adaptive training allows PINN to better satisfy the governing equations by automatically weighing more on large loss terms. 23000 random collocation points, and 4000 uniform boundary points are sampled for $\mathbf{X}_g$ and $\mathbf{X}_b$. The learning rate starts at 0.002 and decays exponentially by a factor of 0.9 for every 1000 epochs, with a total of 10000 epochs. Due to the existence of adaptive weights, all trainable parameters are optimized at full batch using Adam on an NVIDIA A40 GPU.

$$\theta^* = \arg\min_\theta \max_\lambda L(\lambda, \theta) \tag{5}$$

Domain decomposition

Although a smoothed material property domain naturally satisfies the continuity requirement of PINN, the transition region defined by k always generates bias from the real physics solution. To keep the material property precise at the corresponding collocation points, distinct permeability has to be assigned to each material subdomain. In such case, Eq. 4 is not well defined on $\partial\Omega_2$. The discontinuity can be addressed by defining two distinct neural networks $NN_{\theta 1}$ and $NN_{\theta 2}$ for the two domains Ω_1 and Ω_2. Another alternative is to relieve the differentiability requirement through the weak form governing equation, which will be discussed more in *Physics residual formulation*.

When multiple neural networks are defined, the following boundary conditions can be derived using Divergence and Green's Theory (from Eq. 1 and 2) to obtain a unique MVP solution over the entire computation domain:

$$\mathbf{B}_1 \cdot \mathbf{n} = \mathbf{B}_2 \cdot \mathbf{n} \tag{6}$$

$$\mathbf{H}_1 \cdot \mathbf{t} = \mathbf{H}_2 \cdot \mathbf{t} \tag{7}$$

where the normal component of $\mathbf{B}$ and the tangential component of $\mathbf{H}$ should always remain continuous across any material boundary. Fig. S2B illustrates the domain decomposition approach where two different neural networks are used to define the MVP fields on the iron core and vacuum. In this case, the loss function will be computed on $\mathbf{X}_{g1} \subset \Omega_1$, $\mathbf{X}_{g2} \subset \Omega_2$, $\mathbf{X}_{b1} \subset \partial\Omega$, and $\mathbf{X}_{b2}, \mathbf{X}_{b3} \subset \partial\Omega_2$:

$$\begin{cases} L_{g1} = |\nabla_\mathbf{x} \times (\nabla_\mathbf{x} \times NN_{\theta 1}(\mathbf{x})/\mu(\mathbf{x})) - J(\mathbf{x})|^2 \quad \forall \mathbf{x} \in \mathbf{X}_{g1} \\ L_{g2} = |\nabla_\mathbf{x} \times (\nabla_\mathbf{x} \times NN_{\theta 2}(\mathbf{x})/\mu(\mathbf{x})) - J(\mathbf{x})|^2 \quad \forall \mathbf{x} \in \mathbf{X}_{g2} \\ L_{b1} = |\nabla_\mathbf{x} \times NN_{\theta 1}(\mathbf{x}) - [0,1]^T|^2 \quad \forall \mathbf{x} \in \mathbf{X}_{b1} \\ L_{b2} = |\nabla_\mathbf{x} \times NN_{\theta 1}(\mathbf{x}) \cdot \mathbf{n} - \nabla_\mathbf{x} \times NN_{\theta 2}(\mathbf{x}) \cdot \mathbf{n}|^2 \quad \forall \mathbf{x} \in \mathbf{X}_{b2} \\ L_{b3} = |\nabla_\mathbf{x} \times NN_{\theta 1}(\mathbf{x})/\mu(\Omega_1) \cdot \mathbf{t} - \nabla_\mathbf{x} \times NN_{\theta 2}(\mathbf{x})/\mu(\Omega_2) \cdot \mathbf{t}|^2 \quad \forall \mathbf{x} \in \mathbf{X}_{b3} \\ L = \sum_{k \in \{g1,g2,b1,b2,b3\}} \frac{1}{|\mathbf{X}_k|} \sum_{\mathbf{x}^i \in \mathbf{X}_k} \lambda_k^i L_k(\theta, \mathbf{x}^i) \end{cases} \tag{8}$$

where the physics residual is split into two separate loss functions L_{g1} and L_{g2} equipped with distinct relative permeability. The solutions in the two subdomains are matched at the interface $\partial\Omega_2$ through loss functions L_{b2} and L_{b3}, which are equivalent to Eq. 6 and 7 for an infinitesimal area. The entire domain is free of current $J = 0$.

Self-adaptive is again utilized to balance all the loss functions. 20000, 3000 random collocation points are sampled for $\mathbf{X}_{g1}$, and $\mathbf{X}_{g2}$. 4000, 1000 and 1000 uniform boundary points are sampled on boundary $\mathbf{X}_{b1}$, $\mathbf{X}_{b2}$, and $\mathbf{X}_{b3}$. The learning rate starts at 0.002 and decays exponentially by a factor of 0.9 for every 1000 epochs, with a total of 10000 epochs. Here, weight parameters $\theta 1$ and $\theta 2$ are trained simultaneously to minimize the total loss L.

Result comparison

As illustrated in Fig. S3, the physical field solutions from both the property transition and domain decomposition strategies exhibit close conformity with the Finite Element Analysis (FEA) results obtained from COMSOL Multiphysics, when the permeability disparity is only tenfold (Eq. 3 is scaled by a factor of 10). For the case with a smoother transition characterized by a larger transition zone (where $k = 0.3$), there is a noticeable distortion in the $\mathbf{B}$ field near the interface region $\partial\Omega_2$. This is caused by the gradual decrease of permeability along the radial direction. As the transition zone narrows to $k = 0.03$, the resulting solution aligns more closely with both the domain decomposition approach and the FEA outcomes. Across all scenarios, the iron core effectively concentrates and amplifies the magnetic flux density, offering a valid approximation of the actual physical fields when domain property discontinuity is modest.

When the permeability of the iron core is increased significantly to $\mu(\Omega_2) = 1000$, as depicted in Fig. S4, the solutions from the domain decomposition strategy continue to match well with the COMSOL FEA results, even in the presence of a permeability gap of 1000 times. Conversely, the material field smoothing approach leads to disordered magnetic flux vectors within the iron core, diverging from the uniform pattern seen in the FEA results. This discrepancy is largely due to training instabilities, which are direct results of the steep spatial gradient in the transition region (as r approaches r_0) when Eq. 3 is amplified by a factor of 1000. Unlike the property transition method, domain decomposition does not rely on the continuity of properties across the interface $\partial\Omega_1 \cap \partial\Omega_2$. It directly addresses the abrupt property change through the boundary conditions expressed in Eq. 6 and Eq. 7, thereby providing more accurate solutions to the MVP field.

2 Physics residual formulation

The results shown above indicate that domain decomposition provides more accurate physical field solutions compared to domain property smoothing, particularly when there is a significant permeability difference between the iron core and vacuum. Nevertheless, this approach introduces additional complexities in the PINN implementation due to non-differentiable material interfaces. There are two potential methods to mitigate this issue: firstly, applying the strong form governing equation to continuous material subdomains individually and enforcing boundary conditions at the material interfaces; secondly, employing the weak form governing equation across the entire domain Ω, which aligns with minimizing the system's magnetic energy (E_B defined in Eq. 9) as discussed in[4].

$$E_B = \int_\Omega \left(\frac{1}{2\mu}|\mathbf{B}|^2 - JA\right) d\Omega \tag{9}$$

Mathematically, both forms yield the same unique solution. Nevertheless, when used as residual loss of a PINN, they can generate distinct training results depending on problem complexity. The effectiveness of the two residual forms is examined on a 2D C-shape iron core problem (case study one in the main manuscript) as shown in Fig. S5A. The iron core rests in a circular domain $\Omega = \Omega_{in} \cup \Omega_{sc1} \cup \Omega_{sc2} \cup \Omega_{out}$ with a radius of 8. The iron core denoted as Ω_{in} has a thickness of 1 and relative permeability of $\mu(\Omega_{in}) = 1000$, the vacuum domain denoted as Ω_{out} has a relative permeability of $\mu(\Omega_{out}) = 1$, the two source domains share the same permeability as the vacuum domain and each has a current density of $J(\Omega_{sc1}) = -0.5$, $J(\Omega_{sc2}) = 0.5$. Fig. S5B shows the MVP field and the magnetic flux density field calculated by COMSOL FEA, which is assumed to be ground truth.

Strong form

When the strong form governing equations Eq. 1 and 2 are used as PINN residuals directly, two neural networks $NN_{\theta 1} : \Omega/\Omega_{in} \rightarrow R$ and $NN_{\theta 2} : \Omega_{in} \rightarrow R$ are defined to predict the MVP field, one for the iron core and the other

for the vacuum. Therefore, the loss function the point sets $\mathbf{X}_{gin} \subset \Omega_{in}$, $\mathbf{X}_{gout} \subset \Omega_{out}$, $\mathbf{X}_{gsc1} \subset \Omega_{sc1}$, $\mathbf{X}_{gsc2} \subset \Omega_{sc2}$, $\mathbf{X}_{b1} \subset \partial\Omega$, $\mathbf{X}_{b2}, \mathbf{X}_{b3} \subset \partial\Omega_{in}$ are sampled to compute the training loss functions as below:

$$\begin{cases} L_{gout} = |\nabla_{\mathbf{x}} \times (\nabla_{\mathbf{x}} \times NN_{\theta 1}(\mathbf{x})) - \mu(\Omega_{out})J(\mathbf{x})|^2 \quad \forall \mathbf{x} \in \mathbf{X}_{gout} \\ L_{gsc1} = |\nabla_{\mathbf{x}} \times (\nabla_{\mathbf{x}} \times NN_{\theta 1}(\mathbf{x})) - \mu(\Omega_{sc1})J(\mathbf{x})|^2 \quad \forall \mathbf{x} \in \mathbf{X}_{gsc1} \\ L_{gsc2} = |\nabla_{\mathbf{x}} \times (\nabla_{\mathbf{x}} \times NN_{\theta 1}(\mathbf{x})) - \mu(\Omega_{sc2})J(\mathbf{x})|^2 \quad \forall \mathbf{x} \in \mathbf{X}_{gsc2} \\ L_{gin} = |\nabla_{\mathbf{x}} \times (\nabla_{\mathbf{x}} \times NN_{\theta 2}(\mathbf{x})) - \mu(\Omega_{in})J(\mathbf{x})|^2 \quad \forall \mathbf{x} \in \mathbf{X}_{gin} \\ L_{b1} = |NN_{\theta 1}(\mathbf{x})|^2 \quad \forall \mathbf{x} \in \mathbf{X}_{b1} \\ L_{b2} = |\nabla_{\mathbf{x}} \times NN_{\theta 1}(\mathbf{x}) \cdot \mathbf{n} - \nabla_{\mathbf{x}} \times NN_{\theta 2}(\mathbf{x}) \cdot \mathbf{n}|^2 \quad \forall \mathbf{x} \in \mathbf{X}_{b2} \\ L_{b3} = |\nabla_{\mathbf{x}} \times NN_{\theta 1}(\mathbf{x})/\mu(\Omega_{out}) \cdot \mathbf{t} - \nabla_{\mathbf{x}} \times NN_{\theta 2}(\mathbf{x})/\mu(\Omega_{in}) \cdot \mathbf{t}|^2 \quad \forall \mathbf{x} \in \mathbf{X}_{b3} \\ L = \sum_{k \in \{gout, gsc1, gsc2, gin, b1, b2, b3\}} \frac{1}{|\mathbf{X}_k|} \sum_{\mathbf{x}^i \in \mathbf{X}_k} \lambda_k^i L_k(\theta, \mathbf{x}^i) \end{cases} \tag{10}$$

where L_{gsc1}, L_{gsc2}, and L_{gout} in Eq. 10 compute the strong form residual over vacuum with and without current sources. Notice that permeability μ is factored out of the curl operator as it remains constant within each subdomain. L_{gin} computes the strong form residual inside of the iron core subdomain based on the MVP field predicted by $NN_{\theta 1}$. L_{b1} enforces a zero potential on the domain boundary $\partial\Omega$, while L_{b2} and L_{b3} over the material interface $\partial\Omega_{in}$. 30000 random collocation points are sampled for $\mathbf{X}_{out}$, 5000 for $\mathbf{X}_{in}$, and 300 each for $\mathbf{X}_{sc1}$ and $\mathbf{X}_{sc2}$. 6000 uniform boundary points are sampled for $\mathbf{X}_{b1}$, 4000 points for $\mathbf{X}_{b2}$ and $\mathbf{X}_{b3}$. Self-adaptive training is utilized to balance the weights λ in front of different loss functions. The learning rate starts at 0.002 and decays exponentially by a factor of 0.9 for every 1000 epochs, with a total of 10000 epochs.

Weak form

One workaround for domain discontinuity is to adopt the weak form governing equation, whose magnetic energy in Eq. 9 is used as a training loss. Notice that the energy loss has no requirement on the differentiability of permeability μ, and will thus allow the entire MVP field represented by a single neural network NN_θ. Therefore, the weak form PINN training loss can be computed below using sampled point sets $\mathbf{X}_e \subset \Omega$ and $\mathbf{X}_b \subset \partial\Omega$:

$$\begin{cases} L_e = \frac{1}{2\mu(\mathbf{x})} |\nabla_{\mathbf{x}} \times NN_\theta(\mathbf{x})|^2 - J(\mathbf{x}) NN_\theta(\mathbf{x}) \quad \forall \mathbf{x} \in \mathbf{X}_e \\ L_b = |NN_\theta(\mathbf{x})|^2 \quad \forall \mathbf{x} \in \mathbf{X}_b \\ L = \frac{\lambda_e}{|\mathbf{X}_e|} \sum_{\mathbf{x}^i \in \mathbf{X}_e} L_e(\theta, \mathbf{x}^i) + \frac{1}{|\mathbf{X}_b|} \sum_{\mathbf{x}^i \in \mathbf{X}_b} \lambda_b^i L_b(\theta, \mathbf{x}^i) \end{cases} \tag{11}$$

where L_e in Eq. 11 is the Monte-Carlo estimation of magnetic energy[5], and L_b is the same zero potential boundary condition as listed in Eq. 10. The complete loss L is calculated from a weighted sum of L_e and L_b with λ_b determined from self-adaptive training. However, λ_e has a fixed value of 3.3 since the optimal value of L_e is most likely nonzero. A total of 33000 random collocation points are sampled for $\mathbf{X}_e$, and 6000 uniform boundary points for $\mathbf{X}_b$. Other hyperparameters remain unchanged as discussed in the strong form training.

Combined form

In addition to the strong and weak form PINN residuals, we explored a combined formulation incorporating both residual forms within training. Although the two residuals share the same mathematical meaning, they synergistically enhance the training convergence. As the weak energy residual is included, the MVP field can be defined by one neural network NN_θ without enforcing material interface boundary conditions. Therefore, the loss function can be

calculated as below based on sample point sets $\mathbf{X}_g, \mathbf{X}_e \subset \Omega$ and $\mathbf{X}_b \subset \partial\Omega$:

$$\begin{cases} L_g = |\nabla_{\mathbf{x}} \times (\nabla_{\mathbf{x}} \times NN_\theta(\mathbf{x})) - \mu(\mathbf{x})J(\mathbf{x})|^2 \quad \forall \mathbf{x} \in \mathbf{X}_g \\ L_e = \dfrac{1}{2\mu(\mathbf{x})}|\nabla_{\mathbf{x}} \times NN_\theta(\mathbf{x})|^2 - J(\mathbf{x})NN_\theta(\mathbf{x}) \quad \forall \mathbf{x} \in \mathbf{X}_e \\ L_b = |NN_\theta(\mathbf{x})|^2 \quad \forall \mathbf{x} \in \mathbf{X}_b \\ L = \dfrac{\lambda_e}{|\mathbf{X}_e|} \sum_{\mathbf{x}^i \in \mathbf{X}_e} L_e(\theta, \mathbf{x}^i) + \sum_{k \in \{g,b\}} \dfrac{1}{|\mathbf{X}_k|} \sum_{\mathbf{x}^i \in \mathbf{X}_k} \lambda_k^i L_k(\theta, \mathbf{x}^i) \end{cases} \tag{12}$$

Self-adaptive weight parameters λ_g and λ_b are assigned to the strong form and boundary condition residuals, while a constant weight $\lambda_e = 3.3$ is assigned to the weak form energy loss. A total of 33000 random collocation points are sampled for $\mathbf{X}_g$ and $\mathbf{X}_e$, and 6000 uniform boundary points for $\mathbf{X}_b$. Other hyperparameters remain unchanged.

Result comparison

Fig. S6 illustrates the outcomes of the PINN, trained using residuals from strong, weak, and combined forms. The strong form PINN, when implemented with domain decomposition, does not converge correctly to the magnetic fields. Magnetic flux incorrectly appears to traverse the core-vacuum boundary, in contrast to the FEA results in Fig. S5B which correctly show the flux confined within the iron core. This discrepancy could stem from Eq. 6 and 7's local non-uniqueness—where a high magnetic field $\mathbf{B}$ can fulfill the continuity condition by aligning parallel (with a near zero $\mathbf{B}$ field outside) or perpendicular (a zero tangential component) to the core boundary. This creates a locally minimized solution that, while not globally accurate, is favored during neural network training.

Conversely, the weak form PINN yields a notable enhancement, aligning more closely with the COMSOL FEA benchmarks (Fig. S5B) than its strong form counterpart. The energy function's squared flux density term inherently promotes flux confinement within the iron core, bypassing the strong form's erroneous tendencies. However, inconsistencies near the iron core's tips indicate that the solution is not entirely physically valid. Traditional numerical methods like FEA succeed with the minimum energy principle because they employ shape functions with limited differentiability, allowing precise spatial integral calculations via Gaussian quadrature. In contrast, the Monte-Carlo approach approximates the energy integral of an infinitely differentiable neural network NN_θ, which may admit nonphysical creases in the MVP field, evidenced by sudden changes across $\mathbf{x}$ as depicted in Fig. S6.

When both strong and weak form residuals are combined in PINN training, the predicted MVP field and magnetic flux density field are significantly improved as seen in Fig. S6, matching well with COMSOL FEA (Fig. S5B). It is believed that the weak form residual determines the general trend of the MVP field, and the strong form helps to regularize local smoothness.

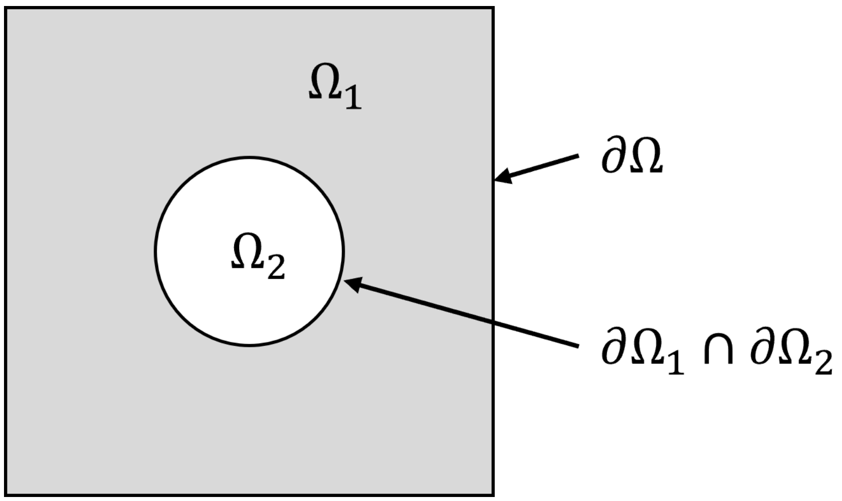

Figure S1. Computation domains and boundaries of a circular iron core in vacuum space.

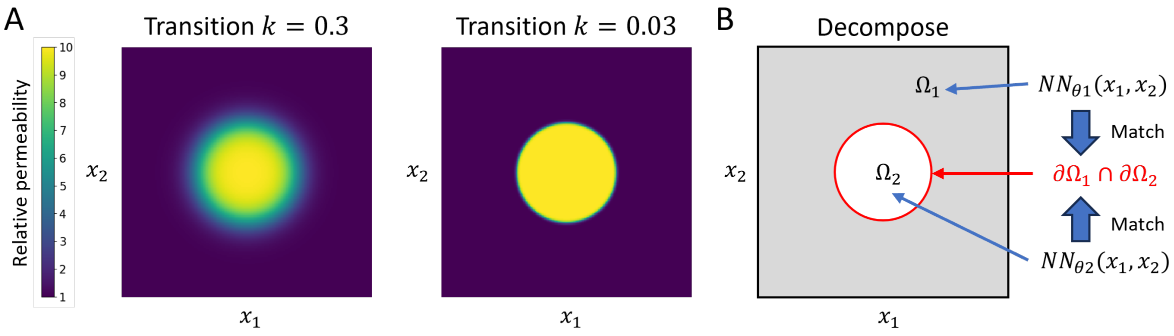

Figure S2. Methods to address discontinuous domain property: (A) Smoothed material domain and (B) Domain decomposition.

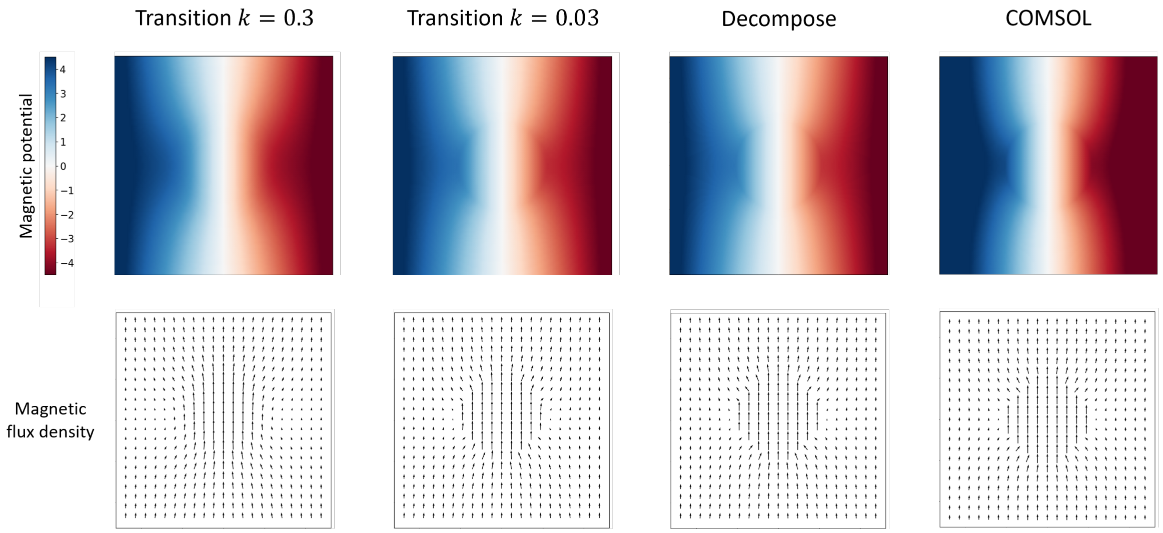

Figure S3. Comparison of the MVP field: A, and magnetic flux density field: $\mathbf{B}$ among PINN solutions for $\mu(\Omega_1) = 1$ and $\mu(\Omega_2) = 10$ using smoothed material domain, domain decomposition, and COMSOL results.

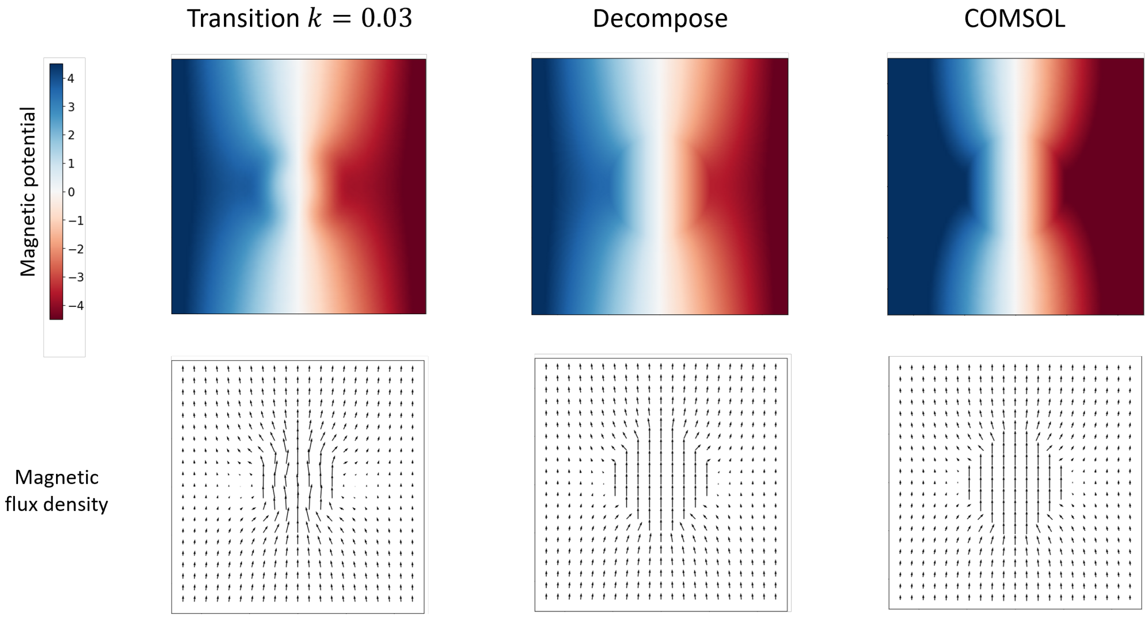

Figure S4. Comparison of the MVP field: A, and magnetic flux density field: $\mathbf{B}$ among PINN solution for $\mu(\Omega_1) = 1$ and $\mu(\Omega_2) = 1000$ using smoothed material domain, domain decomposition, and COMSOL results.

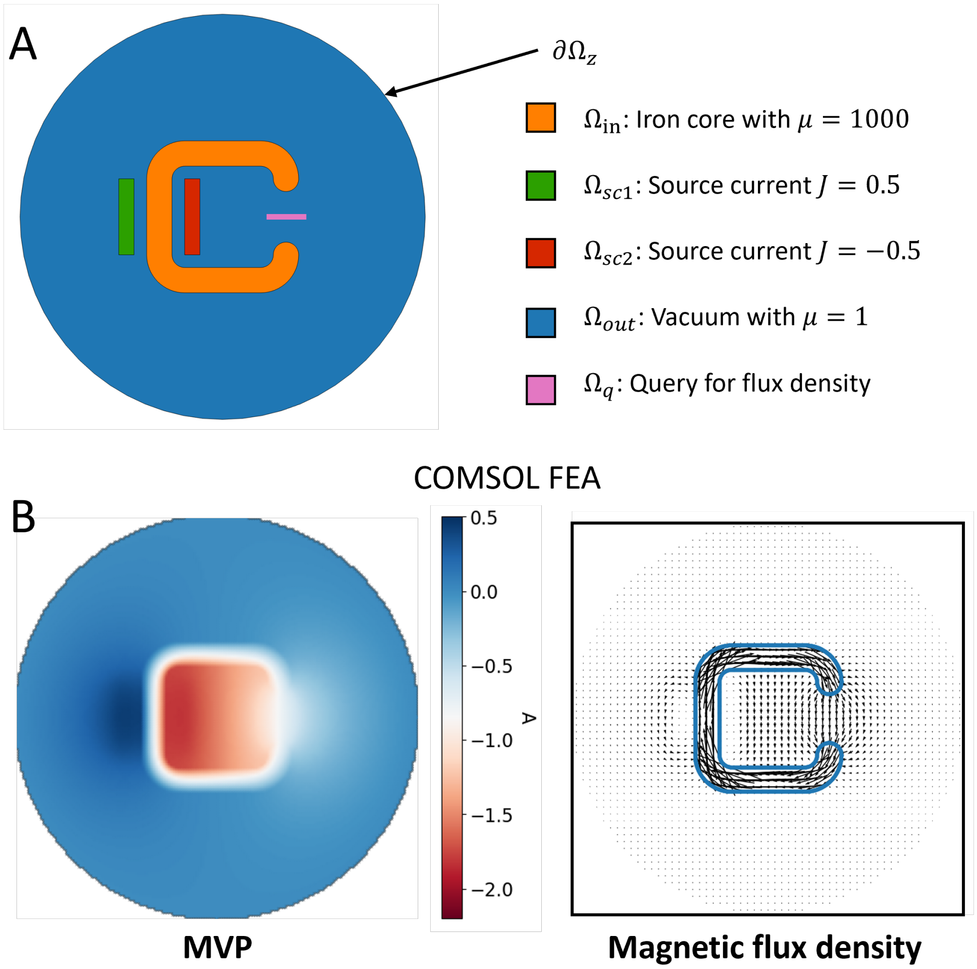

Figure S5. (A) shows the computation domain of the C-shape iron core optimization problem. The MVP field A and magnetic flux density field $\mathbf{B}$ computed numerically by COMSOL FEA is demonstrated in (B).

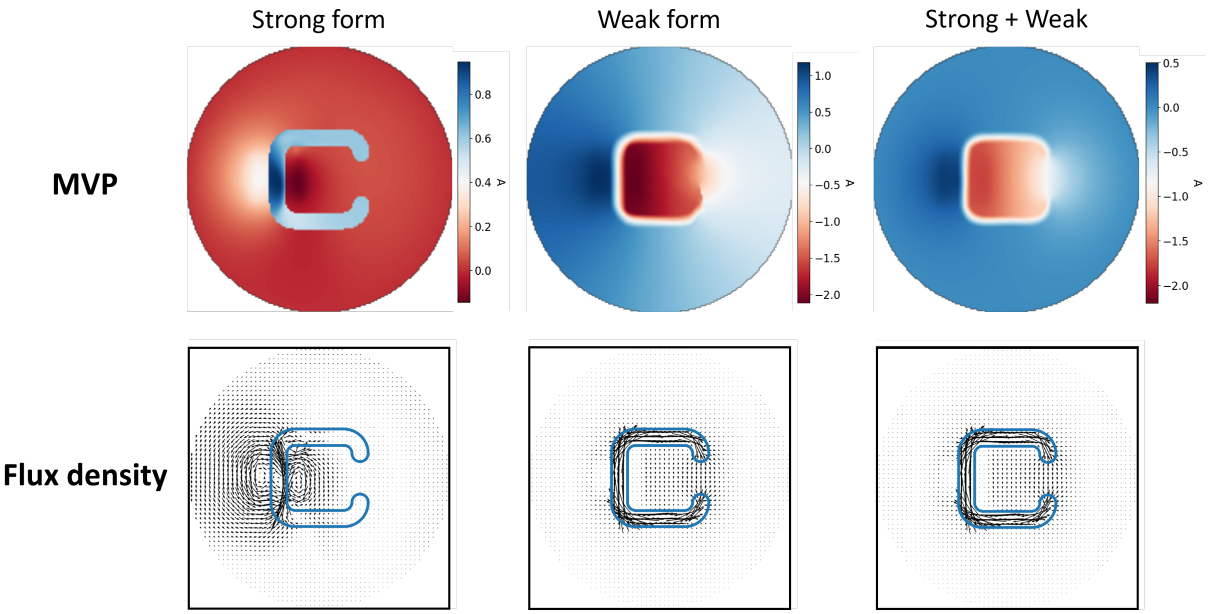

Figure S6. Comparison of the MVP fields A and magnetic flux density fields $\mathbf{B}$ among PINN solutions using the strong form, weak form, and combined form governing equations.

References

1. Jackson, J. D. Classical electrodynamics (1999).

2. Raissi, M., Perdikaris, P. & Karniadakis, G. E. Physics-informed neural networks: A deep learning framework for solving forward and inverse problems involving nonlinear partial differential equations. *J. Comput. physics* **378**, 686–707 (2019).

3. McClenny, L. & Braga-Neto, U. Self-adaptive physics-informed neural networks using a soft attention mechanism. *arXiv preprint arXiv:2009.04544* (2020).

4. Fujiwara, K., Okamoto, Y., Kameari, A. & Ahagon, A. The newton-raphson method accelerated by using a line search-comparison between energy functional and residual minimization. *IEEE transactions on magnetics* **41**, 1724–1727 (2005).

5. James, F. Monte carlo theory and practice. *Reports on progress Phys.* **43**, 1145 (1980).
